# More optimistic treatment expectations are associated with better outcomes through stronger group cohesion, but not dyadic alliance: results from a naturalistic day clinic study in complex depression

**DOI:** 10.3389/fpsyt.2026.1756871

**Published:** 2026-03-05

**Authors:** Catherine Irniger, Johannes Vetter, Steffi Weidt, Erich Seifritz, Martin Grosse Holtforth, Rainer Krähenmann

**Affiliations:** 1 University of Bern, Bern, Switzerland; 2 University of Zurich, Zurich, Switzerland; 3 University Hospital of Psychiatry Zurich, Zurich, Switzerland; 4 University of St. Gallen, St. Gallen, Switzerland; 5 Psychiatry of St. Gallen, Wil, Switzerland; 6 Inselspital Bern, Bern, Switzerland; 7 Psychiatric Services Thurgau, Münsterlingen, Switzerland

**Keywords:** day clinic, depression, dyadic alliance, group cohesion, mediation, naturalistic, treatment expectations, treatment outcome

## Abstract

**Introduction:**

More optimistic treatment expectations are typically associated with better psychotherapy outcomes, including in depression. Meta-analytic findings suggest that this relationship is mediated by the dyadic alliance. In settings including group formats, however, patients also interact and build relationships with one another. Thus, group relationship may represent an important mediator, particularly given that depression is often accompanied by interpersonal difficulties. Therefore, we aimed to investigate the potential mediating role of group relationship above and beyond the dyadic alliance, as, to the best of our knowledge, this has not yet been examined. We hypothesized that more optimistic treatment expectations would be both, directly and indirectly associated with better outcomes through a stronger dyadic alliance and better group relationship.

**Methods:**

Data were drawn from a naturalistic observational study at a local day clinic in Switzerland. Patients were assigned to one of two treatment tracks: Cognitive Behavioral Analysis System of Psychotherapy (CBASP) or Short-Term Psychodynamic Psychotherapy (STPP). Fifty adult patients with complex courses of depression were included in the analysis. Treatment expectations were assessed at baseline using the Treatment Expectation Questionnaire (TEX-Q), dyadic alliance within the first weeks using the Helping Alliance Questionnaire (HAQ), and group relationship with the subscale group cohesion of the Inpatient and Day-Clinic Experience Scale (IDES) retrospectively at treatment end. Treatment outcome was quantified as residual change scores on the revised Beck Depression Inventory (BDI-II) from baseline to treatment end. A parallel mediation analysis controlling for treatment track and duration was conducted in PROCESS using bias-corrected bootstrapping.

**Results:**

More optimistic treatment expectations were significantly associated with higher levels of dyadic alliance and group cohesion. However, only group cohesion was associated with outcome, but not dyadic alliance. The direct association of treatment expectations with outcome was non-significant.

**Discussion:**

These findings provide preliminary support for an indirect association of treatment expectations with outcome through group cohesion in depression. However, the results should be interpreted with caution due to several methodological constraints. Future research should replicate these findings in larger samples, with prospective cohesion measurements, and examine temporal dynamics. Clinically, these findings highlight the potential relevance of explicitly addressing treatment expectations and group cohesion.

## 1 Introduction

In psychotherapy in general (1–4), as well as in depression treatment in specific, more optimistic treatment expectations are typically associated with better treatment outcomes (5, 6). Nevertheless, the pathways through which these expectations affect outcomes, still need further investigation, particularly in depression treatment.

Depression is associated with a pessimistic expectation bias that involves underestimating positive and overestimating negative future events (7–9) and also extends to treatment expectations (10, 11). This bias appears to be rather stable and is often not updated despite disconfirming information (12, 13). Nevertheless, several studies show a mediating effect of the dyadic alliance in the relationship between expectations and outcome in psychotherapy in general (14–17) and in the treatment of depression (18, 19). These findings are supported by meta-analytic findings (20).

In inpatient or day clinic settings, however, patients typically spend a substantial portion of their time with other patients and participate in group therapies (21). Thus, they form relationships not only with the individual psychotherapist but also with other patients and the patient group as a whole (22, 23). Accordingly, meta-analytic findings in mixed patient samples indicate that a better relationship with the group is associated with better treatment outcomes (24, 25). Nevertheless, only a few studies have specifically studied the treatment of depression, with the available evidence also suggesting better outcomes for a better group relationship (26–31). The link between initial treatment expectations and group relationship as well as its potential mediating role in the link to treatment outcome, has, to the best of our knowledge, not yet been examined. However, it is reasonable to assume that treatment expectations in group formats also include expectations about interactions with other patients. This especially applies as depression is typically not only associated with enhanced levels of interpersonal difficulties (32–34) but also with social avoidance and interpersonal fears (35–37).

Thus, the aim of our analysis is to investigate whether group relationship, comparably to dyadic alliance, is a possible linking factor between treatment expectations and treatment outcome in a naturalistic sample of depressed day clinic patients with complex courses of disease, reflecting clinically diverse presentations requiring more intensive care. We hypothesized that treatment expectations and treatment outcome will be partially associated through group relationship. We expected more optimistic treatment expectations to be associated with a stronger dyadic alliance and a better group relationship, which in turn would relate to a better treatment outcome. Furthermore, we expected more optimistic expectations also directly being associated with a better treatment outcome.

## 2 Materials and methods

This analysis was part of the naturalistic, observational study “POP-CD: Process and Outcome in Psychotherapy of Chronic Depression” (38). The study was conducted at the Day Clinic for Depression and Anxiety at the Psychiatric University Hospital Zurich. The study was approved by the local ethics committee (BASEC-No. 2020-00364). Patients were recruited between September 2020 and May 2022. They were referred to the day clinic by local health care professionals, which was the standard of routine care and independent of the study. In an initial pre-treatment interview, patients were informed about the overall structure of the multimodal treatment program, including the two treatment tracks. Furthermore, their eligibility for treatment was assessed by a clinician in line with the day clinic’s treatment focus on adult depression, encompassing a broad range of depressive presentations. In addition, they had to be sufficiently stable and autonomous to attend the treatment program on five days a week. They were not eligible for day clinic treatment if they were at acute risk of harming themselves or others, or exhibited prominent psychotic symptoms or problematic substance use. To participate in the study, they explicitly had to be at least 18 years old and provide written informed consent.

The patients underwent the day clinic treatment in two treatment tracks: Cognitive Behavioral Analysis System of Psychotherapy (CBASP; 39, 40) or Short-Term Psychodynamic Psychotherapy (STPP; 41). Existing findings support evidence for both CBASP (42, 43) and STPP (44–46) in the treatment of depression. However, psychotherapeutic treatment, despite its track-specific focus, was delivered in a flexible, naturalistic manner within tracks. The patients were allocated non-randomly to the tracks as part of routine care procedures. Based on the pre-treatment interviews, the clinicians considered track indication individually, also taking practical aspects such as availability of treatment spots into account. Thus, each track included 10 treatment spots, with patients joining in rolling cohorts. The multimodal treatment program took place five days a week and comprised individual and group psychotherapy, as well as a substantial amount of other components such as occupational therapy, physiotherapy, and daily living training. Approximately 75% of the treatment took place within the track groups, of which three hours per week were dedicated to group psychotherapy. In addition, the patients spent roughly 17% of treatment in the entire patient group which included daily morning briefings and up to two hours of group psychotherapy per week. The group psychotherapies were led by one to two psychotherapists, including focused group discussions and psychoeducation. In addition to the group therapies, the patients received two hours of individual psychotherapy per week. Despite the COVID-19 pandemic, during which a substantial part of the data collection took place, the day clinic was able to resume to and maintain its normal operations after the start of the study, with temporary requirements to wear face masks. On average, the treatment length was about 15 weeks (*SD* = 2.4), ranging from 9.4 to 23.6 weeks, and was comparable across both tracks (see **Table 1**).

**Table 1:** Patient characteristics.

Characteristics		Total sample *n* = 50		CBASP track *n* = 27		STPP track *n* = 23		Group differences
		*n*	%	*n*	%	*n*	%	*p* (statistic)
Gender								.12 (3.6) ^a^
Female		21	42.0	14	51.9	7	30.4	
Male		28	56.0	12	44.4	16	69.6	
Non-binary		1	2.0	1	3.7	–	–	
Age in years (*M, SD*)		37.3	13.1	33.4	13.0	41.9	11.9	.01 (439.5) ^b^
Partnership (yes)		23	46.0	12	44.4	11	47.8	1.00 (0.1) ^c^
Referral								.41 (2.0) ^a^
Inpatient treatment		22	44.0	11	40.7	11	47.8	
Outpatient treatment		22	44.0	14	51.9	8	34.8	
Other treatment		6	12.0	2	7.4	4	17.4	
Previous treatment experience		50	100.0	27	100.0	23	100.0	–
Previous inpatient stay		36	72.0	19	70.4	17	73.9	1.00 (0.1) ^c^
Affective disorder								.62 (2.6) ^a^
F31		2	4.0	1	3.7	1	4.3	
F32		8	16.0	3	11.1	5	21.7	
F33		38	76.0	21	77.8	17	73.9	
F41.2		2	4.0	2	7.4	–	–	
Onset of first depressive episode								.07 (5.5) ^a^
Childhood/adolescence		28	56.0	19	70.4	9	39.1	
Early adulthood		14	28.0	6	22.2	8	34.8	
Mid adulthood		8	16.0	2	7.4	6	26.1	
Years since onset of disease								.33 (6.1) ^a^
≥ 2 weeks,	< 2 years	6	12.0	2	7.4	4	17.4	
≥ 2 years,	< 5 years	5	10.0	3	11.1	2	8.7	
≥ 5 years,	< 10 years	10	20.0	8	29.6	2	8.7	
≥ 10 years,	< 20 years	12	24.0	4	14.8	8	34.8	
≥ 20 years,	< 30 years	7	14.0	4	14.8	3	13.0	
≥ 30		10	20.0	6	22.2	4	17.4	
Comorbid mental disorder(s)		38	76.0	22	81.5	16	69.6	.51 (1.0) ^c^
AD(H)D		7	14.0	4	14.8	3	13.0	1.00 (0.0) ^c^
ANX		15	30.0	8	29.6	7	30.4	1.00 (0.0) ^c^
OCD		5	10.0	2	7.4	3	13.0	.65 (0.4) ^c^
PD/PT		17	34.0	10	37.0	7	30.4	.77 (0.2) ^c^
PTSD		4	8.0	4	14.8	–	–	.12 (3.7) ^c^
SUD		7	14.0	3	11.1	4	17.4	.69 (0.4) ^c^
Other diagnoses		13	26.0	8	29.6	5	21.7	.10 (3.7) ^c^
Comorbid somatic disorder(s)		35	70.0	18	66.7	17	73.9	.76 (0.3) ^c^
AD (yes)		44	88.0	25	92.6	19	82.6	.40 (1.2) ^c^
Treatment length (weeks; *M, SD*)		15.0	2.4	14.8	2.5	15.3	2.3	.52 (343.0) ^b^

F31, Bipolar Disorder; F32, Depressive Episode; F33, Recurrent Depressive Disorder; F41.2, Mixed Anxiety and Depressive Disorder; AD(H)D, attention-deficit (hyperactivity) disorder; ANX, anxiety disorder; OCD, obsessive-compulsive disorder; PD, personality disorder; PT, personality trait; PTSD, post-traumatic stress disorder; SUD, substance use disorder; AD, antidepressant(s); ^a^chi-square test with Monte Carlo estimation (10,000 simulations, 95% confidence interval), exact significance (two-sided); ^b^Mann-Whitney U test, asymptotic significance (two-sided); ^c^chi-square test for 2x2 crosstabulations, exact significance (two-sided).

**Figure 1:**
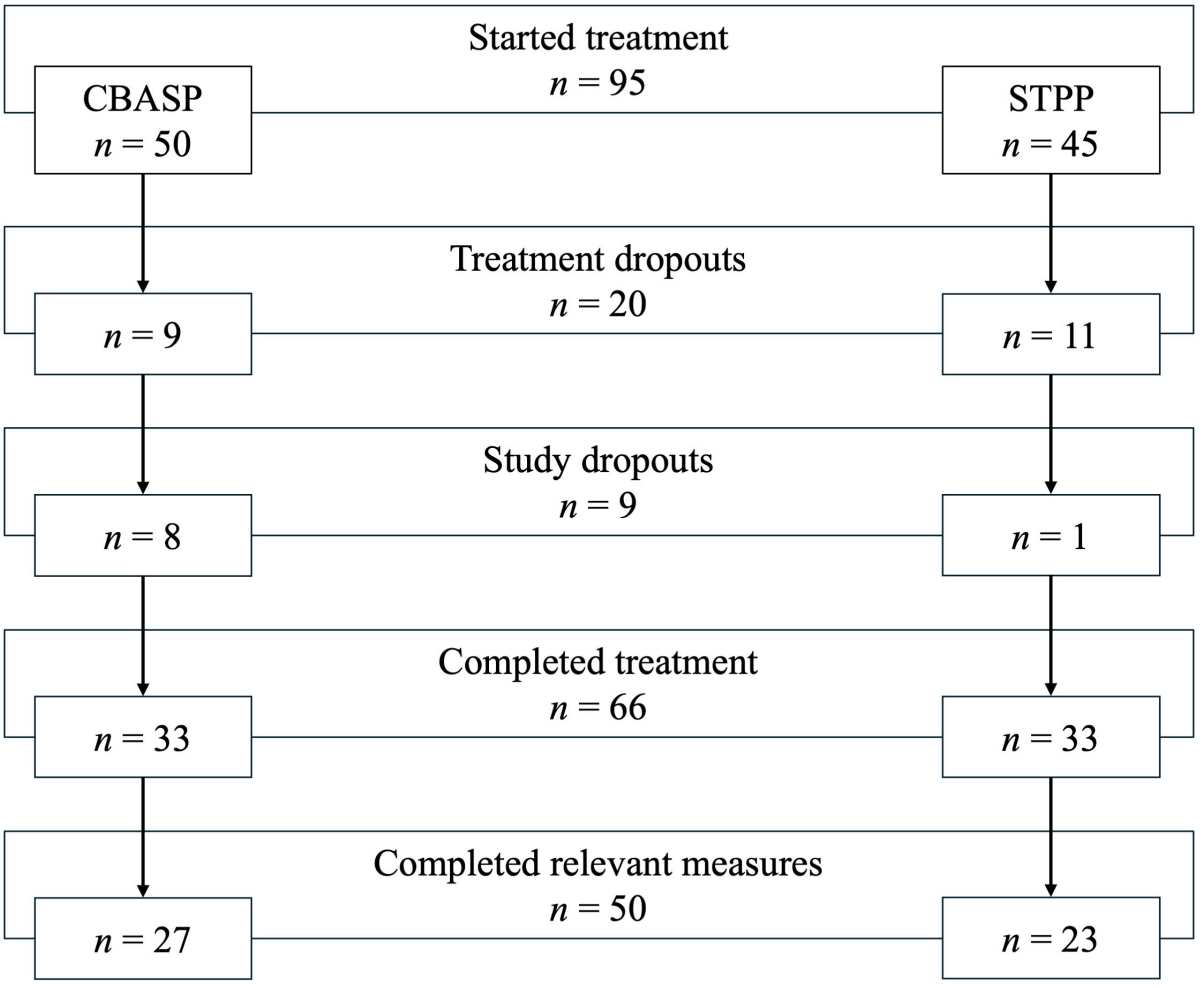
Participant flow.

An overview of the participant flow is presented in **Figure 1**. A total of 95 included patients started their treatment at the day clinic, with a roughly equal distribution across the CBASP and STPP tracks. However, approximately one fifth of patients (*n* = 20) terminated their treatment prematurely, with treatment dropout rates being comparable across both tracks. Reasons for premature treatment termination were transfer to another treatment facility (*n* = 6), perceived lack of treatment fit (*n* = 3), insufficient attendance (*n* = 2), return to work (*n* = 1), lack of insurance approval (*n* = 1), private serious somatic event (*n* = 1), and unknown reasons (*n* = 6). Additionally, about one tenth of patients (*n* = 9) withdrew their study participation and thus were study dropouts, with a notably higher number of patients in the CBASP track. The main reason for study dropout was feeling additionally burdened by the study assessments (*n* = 7), while one patient reported a lack of motivation and another did not provide a specific reason. As can be seen in **Supplementary Table 1**, dropouts descriptively included a slightly higher proportion of female patients and were considerably less often in a partnership. A total of 66 patients completed their treatment, and 50 were included in the present analysis as they completed all relevant measures, with roughly equal representation in each track.

### 2.1 Participants

The detailed sociodemographic and clinic characteristics of the present sample as well as statistical comparisons between tracks can be accessed in **Table 1**. In the total sample, slightly more than half of patients were male, with a higher proportion of women in the CBASP track and a higher proportion of men in the STPP track. However, gender differences did not differ significantly between tracks. On average the patients were about 37 years old with a range of age from 18 to 62 years. The patients in the STPP track were significantly and on average about eight to nine years older than those in the CBASP track. Just under half of patients were in a partnership with a comparable number in both treatment tracks. A similar number of patients was referred by inpatient and outpatient treatment. Although the STPP track had less outpatient referrals compared to the CBASP track, the source of referral did not differ significantly. All patients had prior treatment experience and most of them underwent inpatient treatment before. The majority of patients was diagnosed with a recurrent depressive disorder (F33) according to ICD-10 (47) with an onset of disease in childhood or adolescence and more than ten years ago. Depression onset during childhood or adolescence was more common in the CBASP track, whereas an onset of disease of 10 or more years ago was more frequent in the STPP track. However, statistical differences in onset of disease between tracks were non-significant. Almost all patients were prescribed at least one antidepressant. Most of patients in both tracks showed at least one comorbid somatic or mental disorder with anxiety disorders and personality disorders or traits being most prominent. The frequencies of comorbid mental disorders were comparable in both treatment tracks and did not differ significantly, but PTSD was observed only in the CBASP track.

### 2.2 Measures

*Treatment expectations* were assessed at treatment beginning using the Treatment Expectation Questionnaire (TEX-Q, 48, 49). The TEX-Q is a multidimensional 15-items questionnaire with six subscales. It is a novel and validated questionnaire assessing not only expected benefit from treatment, but also its expected broader impact, potential harms, and anticipated treatment processes. The items are rated on an 11-point Likert-type scale ranging from 0 = *not all* (e.g., no benefit or risk) to 10 = *maximal* (e.g., maximal benefit or risk). The internal consistency for the total mean score was moderate but acceptable with a Cronbach’s Alpha of .69.

*Dyadic alliance* was assessed within the first weeks of treatment by using the subscale *Relation to the Therapist* of the German version of the Helping Alliance Questionnaire (HAQ; 50, 51). The subscale captures the patients’ emotional bond with their individual psychotherapist. It includes six items that are rated on a six-point Likert-type scale ranging from 1 = *strongly disagree* to 6 = *strongly agree*. The internal consistency was good with a Cronbach’s Alpha of .93. Due to the naturalistic setting of the study, the exact timing of administration could not be strictly standardized. On average, the HAQ was administered 2.1 weeks after treatment start (*SD* = 1.0; range 1.0 to 5.9), covering 92% of patients in the second to fourth week of treatment.

*Group relationship* was operationalized as group cohesion and assessed retrospectively at treatment end by using the corresponding subscale of the German version of the Inpatient and Day-Clinic Experience Scale (IDES; 21, 52). The subscale captures the patients’ sense of belonging to the group and the emotional support they receive from it. It includes four items that are rated on a six-point Likert-type scale ranging from 1 = *not true at all* to 6 = *completely true*. The internal consistency was good with a Cronbach’s Alpha of .81.

*Treatment outcome* was assessed using the German version of the revised Beck Depression Inventory (BDI-II; 53, 54) at the beginning (t_0_) and end of treatment (t_1_). The BDI-II includes 21 items which are rated on a 4-point scale ranging from 0 to 3, with higher scores indicating more severe symptoms. The internal consistency for the sum score was good at both measurement time points with a Cronbach’s Alpha of .90 and .94, respectively. Residual change scores were calculated based on the raw score method (55) to quantify symptom change while accounting for baseline levels of depression. Positive values indicate greater improvement than expected and negative values indicate less improvement or deterioration.

### 2.3 Data analysis

All analyses were conducted using a completer-case approach. To test the hypotheses, a parallel mediation analysis was run in SPSS 29 using the PROCESS macro (56). The analysis was controlled for treatment duration and treatment track. Due to the moderate sample size and covariates being entered into the model, bias-corrected bootstrapping with 10,000 samples was used (see 56, 57). Across the sample, only four single items were missing, representing 0.1% of the data, with one missing item for each of four patients. One TEX-Q value was missing and not imputed, as a mean score was calculated. Three BDI-II items at the end of treatment were missing and replaced by the individual time-point mean, as a sum score was computed.

## 3 Results

Descriptive statistics and bivariate correlations for the main study variables are presented in **Table 2** and **3**, with corresponding box plots shown in the **Supplementary Material**. At the beginning of treatment (t_0_), the patients reported depressive symptoms at the upper limit of a moderate level of depression (53), which decreased to the mild-to-moderate range at treatment end (t_1_). Across both treatment tracks, treatment expectations were in the middle range, and dyadic alliance and group cohesion were rated high. Of the relevant study variables, only dyadic alliance and treatment expectations showed a significant bivariate correlation at a moderate level.

**Table 2:** Descriptive statistics of outcome variables and predictors.

Statistic	Depression level t_0_	Depression level t_1_	Treatment expectations	Dyadic alliance	Group cohesion
Total sample					
* M* (*SD*)	26.8 (9.8)	19.8 (11.6)	6.5 (1.0)	29.6 (5.0)	4.8 (0.9)
Range	5.0-45.0	0.0-44.0	4.1-8.8	13.0-36.0	2.5-6.0
Skewness	-0.09	0.3	0.1	-0.9	-0.8
CBASP track					
* M* (*SD*)	27.4 (10.4)	18.6 (11.9)	6.6 (1.0)	30.3 (5.5)	4.7 (1.0)
Range	12.0-45.0	0.0-44.0	4.1-8.8	13.0-36.0	2.5-6.0
Skewness	0.1	0.4	-0.1	-1.4	-0.8
STPP track					
* M* (*SD*)	26.2 (9.2)	21.1 (11.3)	6.5 (1.0)	28.8 (4.3)	5.0 (0.7)
Range	5.0-45.0	2.0-42.5	4.7-8.6	20.0-36.0	3.5-6.0
Skewness	-0.5	0.2	0.3	-0.1	-0.4

t_0_, treatment beginning; t_1_, treatment end; BDI-II, range of 0-63; treatment expectations (TEX-Q), range of 0-10; dyadic alliance (HAQ), range of 6-36; group cohesion (IDES), range of 1-6.

**Table 3:** Zero-order correlation matrix.

Variable	1.	2.	3.
1. Dyadic alliance	–	–	–
2. Group cohesion	.26	–	–
3. Treatment expectations	.28 ^*^	.22	–
4. BDI-II residual change	.14	.26	.09

Spearman’s rho, two-sided testing, ^*^p <.05.

**Figure 2:**
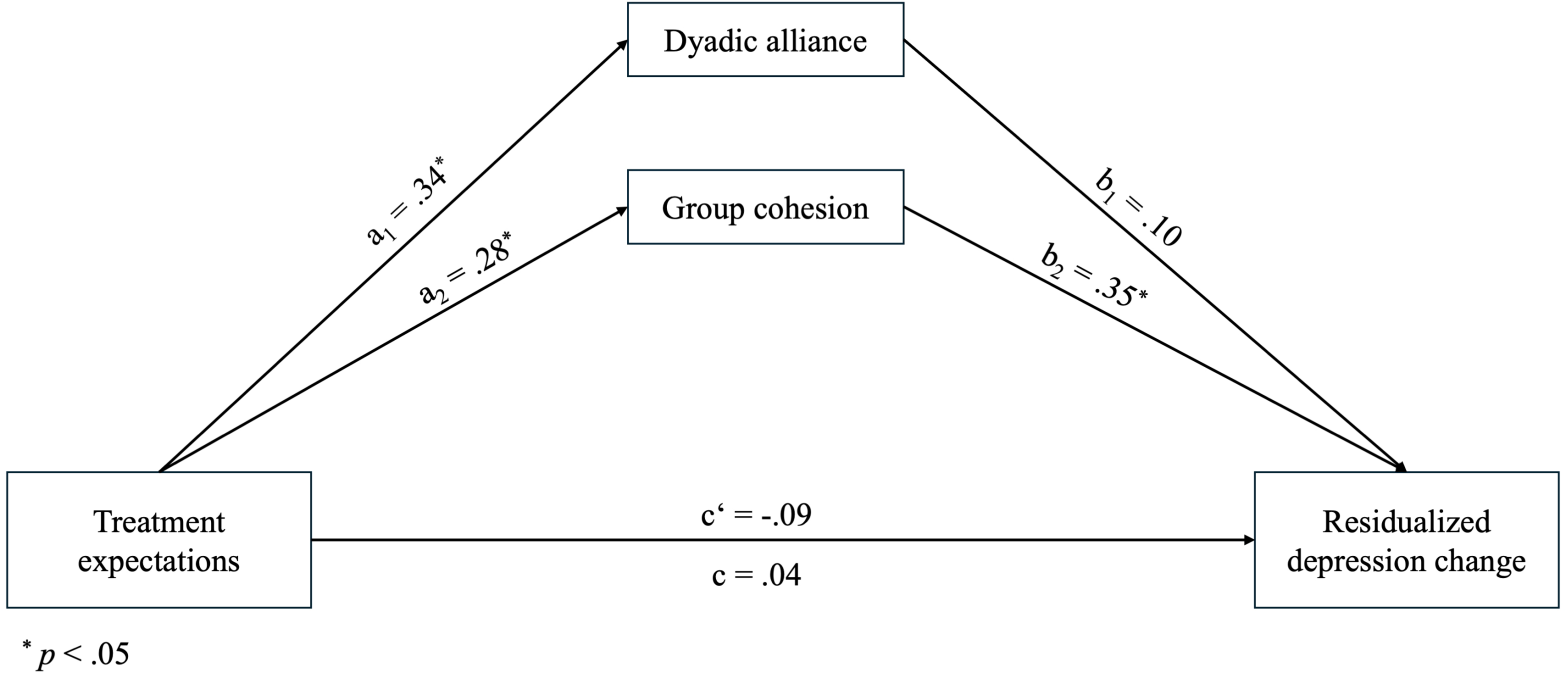
Parallel mediation model with standardized path coefficients.

The main results of the analysis are presented in **Figure 2**, and detailed statistics are provided in **Table 4**. More optimistic treatment expectations were significantly associated with higher levels of dyadic alliance (*β* = .34, *p* = .02) and group cohesion (*β* = .28, *p* = .04). In addition, a longer treatment duration was associated with higher group cohesion (*β* = .31, *p* = .02). Whereas dyadic alliance was not significantly related to treatment outcome (*β* = .10, *p* = .49), group cohesion was (*β* = .35, *p* = .03), with patients in the CBASP track showing more residual change than in the STPP track (*β* = -.29, *p* = .04). Thus, the indirect effect through dyadic alliance was not significant (*β* = .03, 95% CI [-0.25, 1.11]), whereas the indirect effect through group cohesion (*β* = .10, 95% CI [0.06, 2.04]) and the total indirect effect (*β* = .13, 95% CI [0.09, 2.34]) were significant. However, the direct association of treatment expectations with treatment outcome (*β* = -.09, *p* = .55) and the total effect (*β* = .04, *p* = .77) were both non-significant. Overall, the model explained a significant proportion of variance in group cohesion (*R* = .46, *R²* = .21, *F*(3, 46) = 4.04, *p* = .01, *MSE* = 0.67), but not in dyadic alliance (*R* = .37, *R²* = .14, *F*(3, 46) = 2.46, *p* = .08, *MSE* = 22.61) or treatment outcome (*R* = .43, *R²* = .19, *F*(5, 44) = 2.02, *p* = .10, *MSE* = 44.36). All Variance Inflation Factors (VIFs) were within the acceptable range.

**Table 4:** Results of the Mediation Analysis.

Parameter					CI (95%)			
	*b*	*β*	*SE*	*t*	LL	UL	*p*	VIF
Direct effects								
Dyadic alliance								
Treatment expectations	1.67	.34	0.69	2.44	0.29	3.05	.02	1.01
Track	-1.44	-.15	1.36	-1.06	-4.18	1.29	.29	1.01
Length	0.05	.02	0.28	0.17	-0.52	0.62	.87	1.02
Group cohesion								
Treatment expectations	0.25	.28	0.12	2.13	0.01	0.49	.04	1.01
Track	0.23	.13	0.23	0.96	-0.25	0.70	.34	1.01
Length	0.12	.31	0.05	2.36	0.02	0.21	.02	1.02
Depression change								
Treatment expectations	-0.64	-.09	1.05	-0.61	-2.76	1.48	.55	1.21
Dyadic alliance	0.14	.10	0.21	0.69	-0.28	0.56	.49	1.18
Group cohesion	2.77	.35	1.21	2.29	0.33	5.22	.03	1.29
Track	-4.05	-.29	1.95	-2.08	-7.98	-0.12	.04	1.07
Length	-0.23	-.08	0.42	-0.54	-1.07	0.62	.60	1.14
Indirect effects								
Via dyadic alliance	0.24	.03	0.33	–	-0.25	1.11	–	–
Via group cohesion	0.70	.10	0.46	–	0.06	2.04	–	–
Total	0.94	.13	0.55	–	0.09	2.34	–	–
Total effect	0.30	.04	1.00	0.30	-1.72	2.32	.77	-

*b*, unstandardized coefficient; *β*, standardized coefficient; *SE*, standard error; *t*, *t*-value; CI, Confidence Interval; LL, Lower Limit; UL, Upper Limit; *p*, *p*-value; VIF, Variance Inflation Factor.

## 4 Discussion

The analysis revealed a significant indirect association of more optimistic treatment expectations with treatment outcome through higher levels of group cohesion, with treatment track as a significant covariate. However, differences between tracks should be interpreted associational, especially as allocation was not randomized. In addition, other variables such as the significantly higher age in the STPP track, which was not accounted for in the model, as well as unmeasured patient characteristics and therapeutic factors, may have contributed to the observed difference. In contrast to group cohesion, no indirect association through dyadic alliance and no direct association of treatment expectations with treatment outcome was observed. Also, the overall model did not reach statistical significance which, however, was not surprising when considering the multiple non-significant predictors. Thus, only parts of the hypothesized associations were supported, with the results suggesting full instead of partial indirect association through group cohesion but not dyadic alliance. However, the results should be interpreted with caution given the limited sample size, the COVID-19 pandemic conditions, and the missing temporal precedence of group cohesion. However, to the best of our knowledge, this is one of the first studies investigating how group cohesion and treatment expectations in depression therapy relate to each other and to treatment outcome, making the findings an important contribution to the existing literature.

The indirect association of treatment expectations with outcome through group cohesion was consistent with our hypotheses and supports theoretical expectations. This provides novel insight into potential mechanisms connecting treatment expectations and outcome. However, it should be emphasized that no causal conclusions can be drawn from these results. Thus, patients with more optimistic treatment expectations may have engaged more positively with the group, leading to higher levels of group cohesion and thus, a better treatment outcome (35, 58, 59). Alternatively, more optimistic treatment expectations and a higher group cohesion could also be a sign of patients being generally more functional and experiencing fewer social difficulties, which could in turn be associated with treatment outcomes (60–62). However, it should be considered that group cohesion was assessed retrospectively at the end of treatment due to feasibility constraints. Therefore, its association with outcome is likely to be biased, with patients showing more improvement also reporting higher cohesion. In addition, the association between group cohesion and outcome generally grows with more sessions (25), increasing the likelihood of detecting a significant association when it is measured at treatment end. This may also explain why group cohesion was associated with treatment outcome, whereas dyadic alliance was not. Nevertheless, while treatment length was a significant covariate for group cohesion, it was not significantly associated with outcome, although potential moderator effects cannot be ruled out. Furthermore, an exploratory *post-hoc* analysis using the HAQ assessed at treatment end showed no significant association with treatment outcome (see **Supplementary Table 2**). Thus, the lack of an association between dyadic alliance and treatment outcome likely has other explanations and does not appear to be clearly attributable to the different measurement time points.

Nonetheless, it remains surprising that dyadic alliance showed no association with treatment outcome, as many previous studies have reported such a relationship (63, 64). However, previous meta-analytic findings show a medium to large effect for the link between treatment expectations and dyadic alliance, but a small to medium effect for the link between dyadic alliance and treatment outcome (20, 63, 64). Also, a medium effect was observed for the link between group cohesion and treatment outcome (24, 25). This is comparable to the present analysis, which showed a medium effect for group cohesion (*β* = .35) and a small effect for dyadic alliance (*β* = .10). Given the small sample size, the present analysis likely lacked sufficient power to detect the smaller effect of dyadic alliance. Furthermore, some prior studies of dyadic alliance in depression have observed associations only for the “goal and task” component, but not for the “bond” component (65, 66), which was also the component assessed in the present analysis. Additionally, it should be considered that patients in the day clinic treatment setting spend substantially more time with their fellow patients than in individual psychotherapy. Thus, individual psychotherapy is typically only one part of a comprehensive multimodal treatment program. As a result, group cohesion may have played a particularly prominent role. Another possible explanation for the non-significance of dyadic alliance could be that different types of relationships may be relevant for different types of outcomes. Thus, an earlier analysis of data from the same study found that dyadic alliance was linked to quality of life but not self-esteem, whereas group cohesion showed the opposite pattern (30). This suggests that dyadic alliance may be more closely related to global outcomes, whereas group cohesion may be more specifically related to disorder-specific outcomes, such as depressive symptoms. Moreover, all patients had undergone psychotherapeutic treatment before and more than half of patients (58%) reported an onset of disease 10 or more years ago. This may reduce the predictive value of dyadic alliance. Despite mixed findings, some studies report a lower dyadic alliance for individuals with previous treatment experience (67) and a weaker or non-significant association between dyadic alliance and outcome in patients with multiple prior depressive episodes (68–70). In studies on chronic depression, however, an association between dyadic alliance and treatment outcome has been observed (71, 72). Thus, it remains unclear whether the lack of an association in the present study reflects specific characteristics of the sample (e.g., previous treatment experience, course of disease), contextual factors (e.g., treatment setting, COVID-19 pandemic), or other influences not assessed, highlighting the need for further research.

Unexpected results were also observed for treatment expectations. As predicted, more optimistic treatment expectations were associated with a stronger dyadic alliance and higher levels of group cohesion but showed no direct association with treatment outcome. Again, the study may have lacked sufficient power to detect weaker effects as previous research typically reports rather small effects for the association of treatment expectations with outcome (1–3). This may particularly apply as the TEX-Q, unlike many other measures, assesses not only expected improvement, but also potential negative effects of treatment as well as procedural expectations (see 48, 49). Therefore, the overall score may be less strongly associated with symptom change because the measure captures a broader range of expectations. Additionally, the TEX-Q showed an acceptable but lower internal consistency than in the validation study (49; Cronbach’s Alpha = .79), which also may have attenuated associations with outcome. Furthermore, patients with complex courses of depression are likely to show smaller but still clinically meaningful symptom changes (73), which could have reduced variability in outcome and made associations with treatment expectations harder to detect. In line with this, only 13 patients showed a symptom reduction on the BDI-II meeting the criterion for treatment response [i.e., ≥ 50% reduction; e.g., (74)]. However, although no direct association between treatment expectations and outcome was observed, an indirect association through group cohesion was still found. Thus, larger samples are needed to further clarify whether this replicates and represents a partial or full indirect association.

### 4.1 Strengths and limitations

The present study has several strengths and limitations that should be considered when interpreting the findings. One important strength of this study is its naturalistic design, as data were collected in routine care, enhancing ecological validity and generalizability to real-world clinical practice. Additionally, the inclusion of a heterogeneous sample with complex courses of depression provides rare insights into potential pathways linking treatment expectations to treatment outcome. However, the limited sample size reduces the statistical power and robustness of the findings, and thus, the results should be considered preliminary and interpreted with caution. This especially applies when considering the missing temporal precedence of group cohesion. Together with the high number of male patients, especially in the STPP track, and the circumstance that large parts of the study were conducted under COVID-19 conditions, generalizability of the findings is further constrained. Furthermore, the analysis was based on completers only, with many patients who prematurely terminated their treatment that were excluded from the analysis. Especially these patients may have experienced more difficulties during treatment including interpersonal challenges, or may have had low or even unrealistically high expectations (75, 76), which could have introduced attrition bias.

Using residual change scores of depressive symptoms to quantify treatment outcome allowed to control for baseline symptom severity and reduces the risk of regression to the mean. Nevertheless, several measurement limitations constrain the interpretation of the findings. Thus, the internal consistency of the TEX-Q was only moderate and lower than in the validation study (49), which may have reduced the reliability of the findings. Nevertheless, using a multidimensional measure of treatment expectations such as the TEX-Q still seems meaningful, especially in more complex and persistent courses of disease, as it captures a broader range of treatment expectations beyond symptom improvement. Another important limitation is that group cohesion was retrospectively assessed at termination as many patients were notably impaired, which reduced the number of assessments feasible. Therefore, the temporal precedence of group cohesion was not established, and this measure is likely biased by treatment outcome, limiting the interpretability of the findings and the comparability with dyadic alliance. It should also be noted that dyadic alliance and group cohesion are often closely related and not fully independent of each other (77). Thus, the associations cannot be interpreted as the unique contribution of each relationship variable, as shared variance may account for part of the effects. Furthermore, as only one measurement time point of treatment expectations, as well as dyadic alliance and group cohesion was included, their temporal dynamics and the predictive value of later measurements were not taken into account (6, 27, 63, 78).

Overall, despite the limitations, the present analysis offers novel insights into the interplay between relationship variables, treatment expectations, and treatment outcome in the day clinic treatment of complex courses of depression under routine care conditions. While factors such as sample size, retrospective assessment of group cohesion, and single-time-point measurements constrain the interpretation of the findings, the results suggest that in this setting group cohesion may link initial treatment expectations to outcomes.

### 4.2 Implications

The present findings underscore the importance of treatment expectations and group cohesion in day clinic treatment for complex courses of depression, with several implications for future research and clinical practice. Thus, replications under non-COVID conditions, with temporal precedence of group cohesion, and using larger samples, but also in different treatment settings and locations are needed. Future studies should also include more global outcome measures, which can become particularly relevant in complex courses of depression, such as level of psychosocial functioning or quality of life (79, 80).

In addition, it is important to better understand factors contributing to the association of treatment expectations with group cohesion and dyadic alliance, especially for patients being more pessimistic and having enhanced levels of interpersonal difficulties. This may include clinical factors such as symptom severity or chronicity, as well as comorbidities like personality disorders or traits, which were among the most frequent in the current sample. Additionally, other interpersonal factors, including interpersonal style (72, 81), the level of social functioning (82), and prior formative relational experiences, including childhood maltreatment, could be particularly relevant (39, 40, 83, 84). Moreover, it would be valuable to explicitly assess expectations regarding the dyadic alliance (85) and interactions with fellow patients, including both positive but also negative expectations and fears (35, 36). It is reasonable to assume that patients may also influence each other’s expectations. Thus, recent findings suggest that, despite the stability of the pessimistic bias in depression (12), positive reports from other patients could lead to more optimistic treatment expectations (86).

Furthermore, treatment expectations, as well as dyadic alliance and group cohesion, tend to change over the course of treatment (29, 87, 88). Therefore, it would be essential to gain more knowledge about their temporal dynamics but also their interplay with each other and symptom change by using repeated measures and cross-lagged modeling. Accordingly, prior studies show temporal associations between dyadic alliance and expectations (89–91), as well as between relationship variables and outcome (27, 78). However, typically only one type of relationship is included in such studies, and the temporal interplay between treatment expectations and group cohesion has received little attention to date. Nevertheless, we would also expect group cohesion to show temporal dynamics with treatment expectations and outcome variables. Thus, beneficial (92) and potentially harmful (93) social experiences within the patient group are likely to shape expectations (94), which in turn may influence further experiences and treatment outcome. Next to temporal dynamics, it might also provide useful insights to identify patients who improve despite pessimism and low group cohesion, as well as those who do not improve despite optimism and a high group cohesion. Such comparisons could offer new starting points to inform interventions aimed at fostering adaptive treatment expectations and supporting patients with interpersonal difficulties when interacting with fellow patients.

In clinical practice, integrating expectation- and relationship-focused interventions on the individual and group level may be promising. Thus, addressing patients’ positive and negative treatment expectations at treatment beginning appears essential, including expectations regarding relationships with both the individual therapist and the patient group. Additionally, interventions that foster group cohesion could help to improve outcome (25), which could be particularly important for patients with pessimistic expectations but also for those with interpersonal difficulties. Such interventions may also include sharing positive experiences (86), which could be associated not only with more optimistic expectations, but also help patients with interpersonal difficulties to build trust and encourage them for behavior change. Moreover, explicitly discussing relational processes and expectations within the patient group, both on a meta level and on a process level, may be an important component to foster awareness and support a constructive approach to them. Taken together, these considerations highlight potential ways to translate the findings into clinical practice. However, these implications should be considered with caution as no causal conclusions can be drawn from the results and further research is needed.

## 5 Conclusion

This study provides preliminary evidence that treatment expectations may be associated with treatment outcome through group cohesion in depressed day clinic patients. Although more optimistic expectations were associated with both a stronger dyadic alliance and higher group cohesion, only group cohesion was associated with treatment outcome. These findings underscore the potential relevance of social relationship factors in the treatment of depression. Future research should replicate these findings in larger samples, with temporal precedence of the mediator, and examine temporal dynamics to clarify the directionality of associations. Clinically, the results point to the potential relevance of addressing treatment expectations and fostering group cohesion as aspects that may support therapeutic engagement in patients with depression.

## Data Availability

The datasets presented in this article are not readily available because it includes highly sensitive health data from a highly specific time and location, and the full information provided by the patients could lead to identification. Requests to access the datasets should be directed to psCTC@bli.uzh.ch. Please note, access will be subject to institutional discretion.
