# Supplementary material for: More optimistic treatment expectations are associated with better outcomes through stronger group cohesion, but not dyadic alliance: results from a naturalistic day clinic study in complex depression
Source: Front Psychiatry. 2026 Mar 5;17:1756871. doi: 10.3389/fpsyt.2026.1756871 (PMC12999886; doi:10.3389/fpsyt.2026.1756871)
Supplement: Supplementary file 1 [file Supplementaryfile1.pdf]

Supplementary Material

Supplementary Table 1

Basic Demographic Characteristics of Dropouts

	all dropouts *n* = 29		treatment dropouts *n* = 20		study dropouts *n* = 9	
	n	%	*n*	%	*n*	%
Gender						
Female	15	51.7	10	50.0	5	55.6
Male	14	48.3	10	50.0	4	44.4
Non-binary	-	-	-	-	-	-
Age in years (*M, SD*)	39.1	11.7	38.9	12.9	39.7	9.3
Partnership (yes)	7	24.1	4	20.0	3	33.3

Supplementary Figure 1

Box Plot of Depression Level t_0

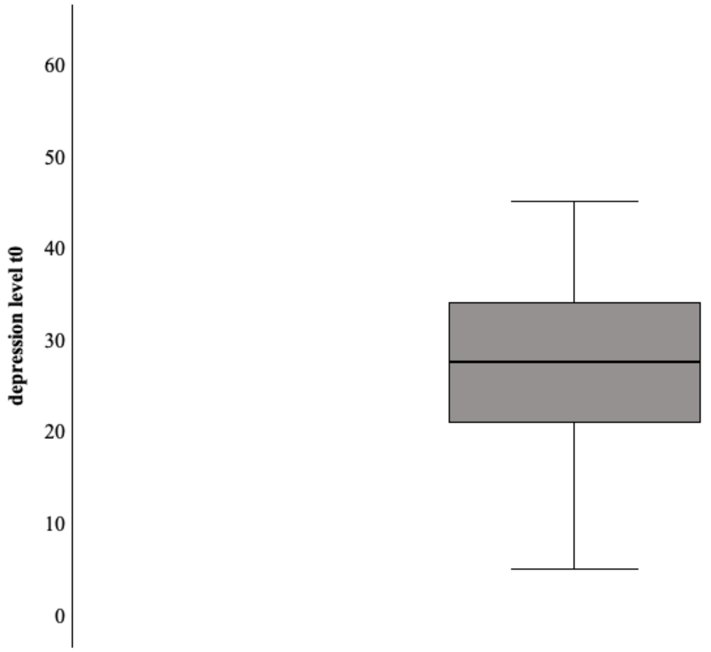

Supplementary Figure 2

Box Plot of Depression Level t_1

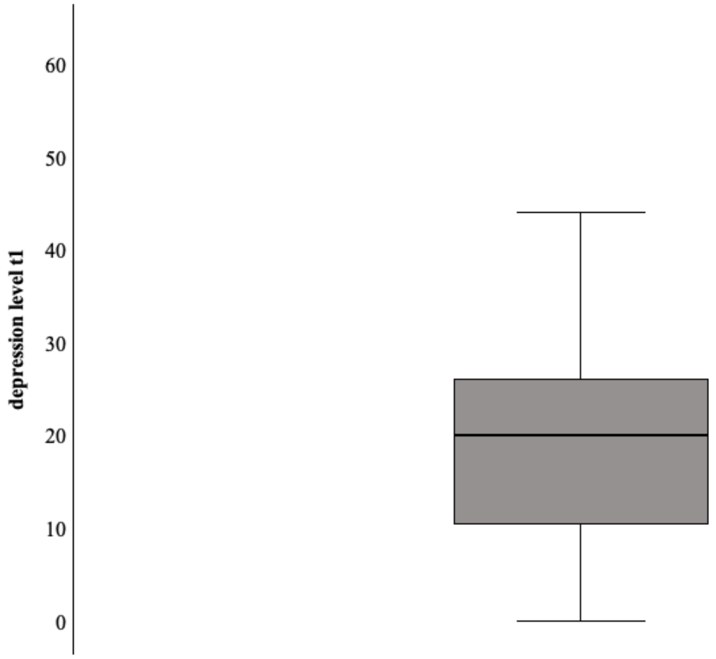

Supplementary Figure 3

Box Plot of Treatment Expectations

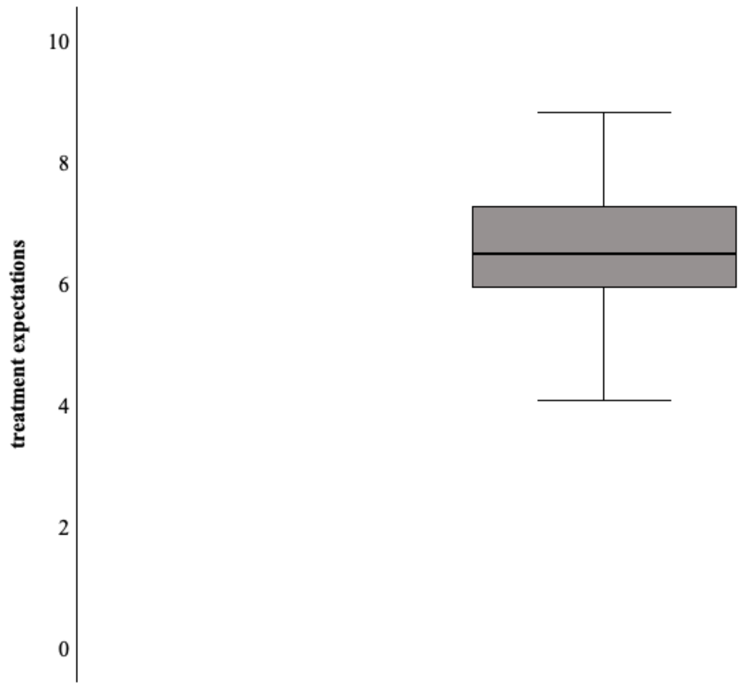

Supplementary Figure 4

Box Plot of Dyadic Alliance

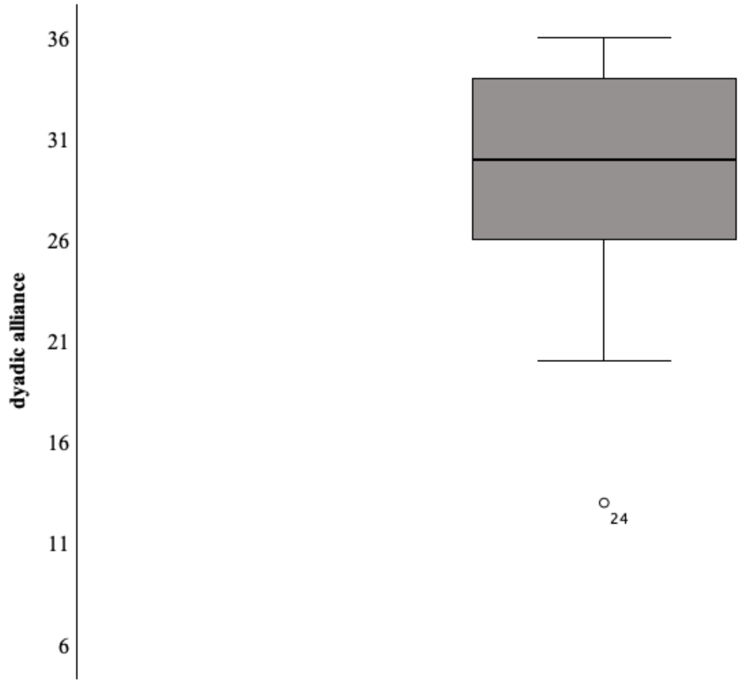

Supplementary Figure 5

Box Plot of Group Cohesion

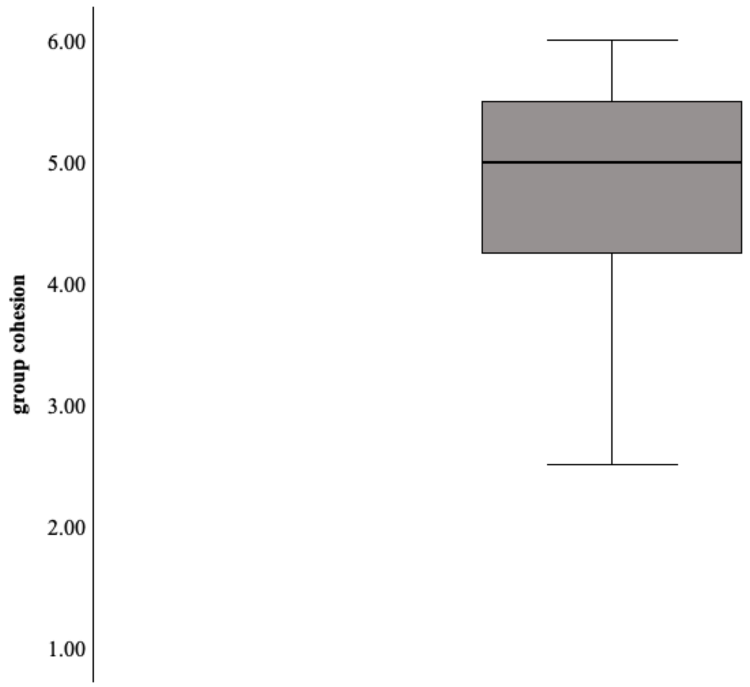

Supplementary Table 2

Results of the Mediation Analysis with Dyadic Alliance Assessed at Treatment End (t_1)

	b	*β*	*SE*	*t*	CI (95%) LL	CI (95%) UL	*p*	VIF
Direct effects								
Dyadic alliance (t_1)								
Treatment expectations	0.76	.18	0.59	1.29	-0.42	1.95	.20	1.01
Track	-1.40	-.17	1.17	-1.20	-3.75	0.95	.24	1.01
Length	0.25	.15	0.24	1.04	-0.24	0.74	.30	1.02
Group cohesion								
Treatment expectations	0.25	.28	0.12	2.13	0.01	0.49	.04	1.01
Track	0.23	.13	0.23	0.96	-0.25	0.70	.34	1.01
Length	0.12	.31	0.05	2.36	0.02	0.21	.02	1.02
Depression change								
Treatment expectations	-0.44	-.06	1.02	-0.43	-2.50	1.61	.67	1.12
Dyadic alliance (t_1)	0.03	.02	0.25	0.12	-0.47	0.52	.91	1.13
Group cohesion	2.86	.36	1.23	2.33	0.39	5.33	.02	1.31
Track	-4.24	-.31	1.97	-2.15	-8.21	-0.27	.04	1.08
Length	-0.24	-.08	0.42	-0.56	-1.09	0.62	.58	1.15
Indirect effects								
Via dyadic alliance (t_1)	0.02	.00	0.17	-	-0.26	0.43	-	-
Via group cohesion	0.72	.10	0.48	-	0.06	2.08	-	-
Total	0.74	.10	0.50	-	0.03	2.09	-	-
Total effect	0.30	.04	1.00	0.30	-1.72	2.32	.77	-

Note. t_1 = treatment end, *b* = unstandardized coefficient, *β* = standardized coefficient, *SE* = standard error, *t* = *t*-value, CI = Confidence Interval, LL = Lower Limit, UL = Upper Limit, *p* = *p*-value, VIF = Variance Inflation Factor.
